# Prevalence of iron-deficient but non-anemic university athletes in Japan: an observational cohort study

**DOI:** 10.1080/15502783.2023.2284948

**Published:** 2023-11-29

**Authors:** Takahiro Nabeyama, Yosuke Suzuki, Hiroaki Saito, Kana Yamamoto, Michiko Sakane, Yoichiro Sasaki, Haruka Shindo, Morihito Takita, Masahiro Kami

**Affiliations:** a University of Tsukuba, Faculty of Health and Sport Sciences, Tsukuba, Ibaraki, Japan; b Medical Governance Research Institute, Minato-ku, Tokyo, Japan; c Department of Internal medicine, Soma Central Hospital, Soma, Fukushima, Japan; d Sakane M Clinic, Tsukuba, Ibaraki, Japan; e University of Tsukuba, Graduate School of Comprehensive Human Sciences, Tsukuba, Ibaraki, Japan; f Navitas Clinic, Department of Internal Medicine, Tachikawa, Tokyo, Japan

**Keywords:** Iron deficiencies, sports nutritional sciences, adolescent health

## Abstract

**Background:**

Iron deficiency (ID) and iron deficiency anemia (IDA) are long-standing health problems in athletes, affecting both performance and health. ID prevalence in young athletes remains high and a matter of concern. ID and IDA can lead to fatigue, reduced endurance, and decreased oxygen transport, potentially compromising athletic performance. We hypothesized that ID would still be a major health concern in university athletes across sports clubs in Japan.

**Purpose:**

The study aimed to investigate the prevalence of ID and IDA in athletes participating in Kendo, badminton, baseball, and handball at the University of Tsukuba (Tsukuba, Ibaraki Prefecture, Japan). The study also examined the correlation between hypoferritinemia and other variables, such as previous use of iron supplements, body mass index (BMI), energy intake, and years of athletics.

**Methods:**

Between January and December 2019, 126 university athletes, consisting of 79 males and 47 females, underwent physical measurements and blood tests. The blood test included complete blood count, levels of serum ferritin, serum iron, and total iron-binding capacity. The anemia was defined in accordance with the WHO criteria. Daily energy and iron intake were estimated with the food frequency questionnaire in Japanese (FFQg). Thirty-four female athletes responded to a survey about their menstruation and low-dose estrogen-progestin (LEP) usage.

**Results:**

While none of the athletes had anemia, 22 (47%) female athletes exhibited serum ferritin levels of 30 ng/mL or less, defining them as hypoferritinemia. The multivariate logistic regression model revealed that a shorter duration of the athletic experience (adjusted odd ratio [95% confidence interval]: 0.62 [0.43–0.90]), lower energy intake (0.994 [0.989–0.999]), and higher dietary iron intake (4.40 [1.12–17.26]) were associated with hypoferritinemia. Seventeen (50%) female athletes reported a decline in subjective performance during menstruation, albeit two took LEP regularly.

**Conclusions:**

This study reveals that ID is a prevalent health concern among young female athletes across sports clubs. It underscores the need for their education on the importance of assessing ID status. Limitation includes the nature of single-site and observational study, the absence of hepcidin measurement, and an unspecified amount of exercise. Comprehensive investigations are needed to elucidate the causes and optimal treatments for ID in young athletes.

## 1. Introduction

Iron deficiency (ID) and iron deficiency anemia (IDA) have been a long-standing health problem in athletes [1,2]. Several mechanisms contribute to developing the ID status and IDA in athletes. One primary mechanism is insufficient iron intake, which can be exacerbated by the increased demand of athletic training [3]. Additionally, athletes may experience iron malabsorption in the gut due to factors like inflammation, which is often a result of intense physical activity [4]. Inflammation can increase levels of hepcidin, a key regulator of iron homeostasis. Elevated hepcidin levels reduce iron absorption in the gut and hinder iron release from cells [5,6]. Furthermore, certain athletic practices, such as high-altitude training, can affect iron levels by increasing red blood cell production, leading to increased iron demand. Low-energy availability, often seen in endurance athletes or those in weight-class sports, can also contribute to reduced iron absorption [7,8].

The nutritional approaches to iron supplementation in athletes have been investigated to maintain sufficient iron storage [9]. Oral iron supplements are commonly used for this purpose. They’re affordable and practical but can cause gastrointestinal issues. Their absorption can be influenced by diet. Despite these challenges, they are typically effective for mild to moderate deficiencies or non-anemic athletes [10]. Intravenous (IV) iron, which bypasses the gut, quickly boosts iron levels. It’s ideal for those intolerant to oral supplements or with severe deficiencies. Even for iron-deficient non-anemic athletes, IV iron contributes to improving iron metabolism [11]. However, IV iron supplementation is more invasive and has risks, including potential iron overload.

ID has been a notable health concern among young athletes. Nicotra et al. reported that 63% and 89% of male and female athletes, aged 11–18 years and affiliated with ball game clubs, exhibited ID or IDA. This study defined the ID as below 30 µg/L of serum ferritin level [12]. Similarly, Sims et al. demonstrated that 46% of nonprofessional but internationally competitive female endurance athletes were ID with less than 30 µg/L of serum ferritin level [13]. Menstrual blood loss, body mass index (BMI), were associated with ID and IDA in young athletes [14]. Certain high-intensity sports can result in increased hepcidin levels [15,16]. Early intervention can prevent declines in hemoglobin for young athletes with ID and IDA; however, the exact threshold for treating hypoferritinemia in young athletes remains unestablished [9]. While there are advantages, such as sustained performance, the potential risk of iron overload warrants careful consideration.

It is not yet known about levels of hypoferritinemia to be treated in young athletes with regard to improving athletic performance [17]. Iron supplementation could be more beneficial in individuals with severe hypoferritinemia. Some placebo-controlled clinical studies evaluating the effect of iron supplementation demonstrated preservation or improvement in exercise capacity in the intervention group in young female athletes [18–21]. However, these were small-size studies, which require justification with a large cohort. Of note, iron supplementation has been found to have beneficial effects on the degree of fatigue and mood disorders in addition to training efficiencies [22].

In this context, it is relevant to examine the prevalence of hypoferritinemia in young university athletes and identify the contributing factors. We showed the prevalence of ID in the university *Kendo* practitioners aged between 18 and 23 years in Japan, where 41% of females, but none of the males, were ID [23]. The high prevalence of ID in female athletes suggests that ID is an underrecognized and unsolved health issue for Japanese university athletes. Most athletes in Japan, with the exception of very few professional athletes, have not been assessed for ID status and are not adequately supplemented with iron. The announcement by the Japanese Athletes Federation indicating the risk of iron injections in 2018 might cause them to fear iron overload [24]. No recent study was published investigating the prevalence of ID in young athletes in Japan. In the outpatient clinic setting, Yamamoto et al. reported a retrospective chart review evaluating athletes aged 13–22 years, where hypoferritinemia was observed in 21% and 43% of males and females, respectively [25].

We hypothesized that the ID would continue to be a primary health concern in university athletes across sports clubs in Japan, as our previous study [23]. Herein, we extend the observation study to include university athletes of badminton, baseball, and handball players.

## 2. Methods

### 2.1. Participants

Between January and December 2019, we recruited the study participants from the badminton, baseball, handball, and kendo clubs at the University of Tsukuba. Prior to participation, each athlete provided written informed consent. There were no specific inclusion criteria unless the candidates disagreed with the participation of the study. Blood tests, physical measurements, and a nutritional survey were conducted. All evaluations were performed by a Board-Certified Sports Medicine physician of the Japan Sports Association (Sakane M Clinic, Tsukuba, Ibaraki, Japan). The Institutional Review Board of the Medical Governance Research Institute (Tokyo, Japan) approved this study (approval Number; MG2018–12).

### 2.2. Measurements

Physical measurements of height and body weight were performed in an outpatient clinic (Sakane M Clinic, Tsukuba, Ibaraki, Japan). Body fat percentage was measured with a BC-118D body composition analyzer (Tanita Corp, Tokyo, Japan). The peripheral venous blood was collected at rest and shipped to the contract laboratory (SRL, Inc., Tokyo, Japan). The physical measurement and blood collection were conducted during break time after lunch to maintain consistent conditions for all participants. The complete blood count, levels of serum ferritin, serum iron, and total iron-binding capacity (TIBC) were measured. Dietary energy and iron intakes were estimated using a food-frequency questionnaire based on food groups (FFQg) [26]. The FFQ is a widely used dietary assessment tool that assesses an individual nutrient intake in a specified period. In this study, we utilized an FFQ tailored to the Japanese population’s dietary habits, making it one of the most commonly used questionnaires for dietary surveys in Japan (Supplementary file). Participants were provided with individual questionnaires to record the frequency and portion sizes of various food items they consumed. This method provides a comprehensive overview of dietary habits, allowing for the estimation of both energy and specific nutrient intakes, including iron. In addition to the FFQg, we asked about their histories of anemia and iron supplement intake. The brief questionnaires about menstruation and usage of low-dose estrogen-progestin (LEP) were administered to the female athletes to determine the association between ID and menstruum management.

### 2.3. Definitions

Anemia was defined as being a hemoglobin level of <13 g/dL for men or less than 12 g/dL for non-pregnant women, in accordance with the World Health Organization (WHO) criteria [27]. We stratified by several cutoff values of serum ferritin level (15, 30, 50, and 100 µg/L) in descriptive statistics since multiple values have adopted hypo-ferritinemia thresholds in past studies [28,29]. We employed a threshold of 30 µg/L to distinguish between hypo- and normo-ferritinemia in the comparison analysis for both male and female participants.

### 2.4. Statistical analysis

The participant and hematological characteristics were summarized using descriptive statistics. The two-group comparison was determined with the Mann-Whitney U test or Fisher’s exact test. The three-group comparison was determined with the Kruskal-Wallis test. Univariate regression followed by multivariate logistic regression analyses were performed to reveal the factors associated with hypoferritinemia. We included age, experience years of the sports, type of the sports club, history of anemia, BMI, and intakes of iron and energy as explanatory variables in the regression models. All the statistical analyses were performed using Microsoft Excel 2016, and the modified version of R (The R Foundation for Statistical Computing, Vienna, Austria) called EZR (Saitama Medical Center, Jichi Medical University, Saitama, Japan) [30], which allows a graphical user interface. *P* values < 0.05 were considered as statistical significance.

## 3. Results

### 3.1. Participant characteristics

A total of 126 university athletes, consisting of 79 males and 47 females, participated in this study. Participant characteristics were summarized with a classification of sex (Table 1). Seven athletes (6% of all participants, a male and six female) had a history of medically diagnosed anemia. Twenty (25%) males and twenty (43%) females took oral iron supplements. Of note, no females in this study were pregnant. The median [interquartile range] of daily energy intake estimated from FFQg was 2,297 [1,842 ‒ 2,809] kcal and 1,916 [1,629 ‒ 2,322] kilocalories (kcal) in males and females, respectively, and the iron intake was 7.2 [5.0 ‒ 8.8] mg and 6.2 [5.0 ‒ 8.0] mg. Significant differences between the sex groups were observed in height (*p* < 0.001), body weight (*p* < 0.001), BMI (*p* < 0.001), history of anemia (*p* = 0.007), and daily energy intake (*p* = 0.012).

**Table 1.** Participant characteristics.

Variables	Total (*n* = 126)	Male (*n* = 79)	Female (*n* = 47)
Age (years)	20 [19 ‒ 21]	20 [19 ‒ 21]	19 [19 ‒ 21]
Years of sports/athletics	13 [11 ‒ 15]	14 [12 ‒ 15]	12 [8 ‒ 15]
Affiliated club			
Badminton	20 (16)	13 (16)	7 (15)
Baseball	21 (17)	21 (27)	0 (0)
Handball	20 (16)	0 (0)	20 (43)
Kendo	65 (52)	45 (57)	20 (43)
Height (cm)	171 [165 ‒ 177]	174 [171 ‒ 179]	163 [158 ‒ 166]
Body weight (kg)	72 [65 ‒ 80]	75 [69 ‒ 83]	62 [57 ‒ 66]
Body mass index (kg/m^2^)	24.4 [23.2 ‒ 25.8]	24.6 [23.7 ‒ 26.1]	23.1 [21.8 ‒ 24.3]
Body fat percentage (%)^†^	16.3 [12.7 ‒ 20.9]	14.3 [12.1 ‒ 17.3]	24.7 [22.0 ‒ 27.8]
History of anemia^†^	7 (6)	1 (1)	6 (13)
Previous use of iron supplement^†^	40 (32)	20 (26)	20 (44)
Food Frequency Questionnaire			
Total energy intake (kcal/day)	2,151 [1,736 ‒ 2,707]	2,297 [1,842 ‒ 2,809]	1,916 [1,629 ‒ 2,322]
Iron intake (mg/day)	6.7 [5.0 ‒ 8.6]	7.2 [5.0 ‒ 8.8]	6.2 [5.0 ‒ 8.0]

Median [interquartile range] or number (percentage) are shown. ^†^ Missing data -total number (male/female); 21 (1/20), 3 (2/1) and 4 (3/1) in body fat percentage, history of anemia and previous use of iron supplement, respectively.

### 3.2. Prevalence of iron deficiency

No participants were identified as having anemia defined by the WHO criteria. In the evaluation of iron status, a total of 22 (17% of all cohort, 95% confidence interval [CI]: 11–25%) participants exhibited ferritin levels of 30 ng/mL or less, which is defined as hypoferritinemia in this study (Table 2). Hypoferritinemia was observed solely in females (47%, 95%CI: 32–62%) and none of the male athletes.

**Table 2.** Anemia and iron status.

Variables	Total (*n* = 126)	Male (*n* = 79)	Female (*n* = 47)
Anemia status			
Prevalence of anemia*	0 (0)	0 (0)	0 (0)
RBC (×10^6^/μL)	495 [454–522]	510 [493.5–540.5]	444 [425–471.5]
Hemoglobin (g/dL)	14.8 [13.8–15.5]	15.3 [14.75–15.85]	13.6 [12.65–14.1]
Hematocrit (%)	45.1 [42.8–47.4]	46.8 [44.8–48.35]	42.2 [39.9–44.15]
MCV (fL)	92 [90–94]	91 [89–93]	94 [92–96]
MCH (pg)	30.1 [29.2–30.7]	29.9 [29.2–30.4]	30.7 [29.7–31.05]
MCHC (g/dL)	32.6 [31.9–33.1]	32.8 [32.4–33.3]	32.1 [31.65–32.7]
Iron Status			
Serum iron level (μg/dL)	89.5 [67–130]	93 [67–133]	88 [67–126]
TIBC (μg/dL)	338.5 [318–362]	331 [318.5–351]	347 [322–387.5]
Prevalence of hypoferritineamia −*n* (%, 95% CI)	22 (17, 11–25)	0 (0, 0–5)	22 (47, 32–62)
Ferritin (µg/L)	70 [31-]105]	94 [70–128]	35 [20-]49]
>100 and ≤ 300	40 (32)	38 (48)	2 (4)
>50 and ≤ 100	39 (31)	31 (39)	8 (17)
>30 and ≤ 50	25 (20)	10 (13)	15 (32)
>15 and ≤ 30	14 (11)	0 (0)	14 (30)
≤15	8 (6)	0 (0)	8 (17)

Median [interquartile range] or number (percentage) are shown. Abbreviations: RBC. red blood cell; MCV, mean corpuscular volume; MCH, mean corpuscular hemoglobin; MCHC, mean corpuscular hemoglobin concentration; TIBC, iron binding capacity. *Definition of anemia is accordance with WHO criteria.

**Figure 1.**
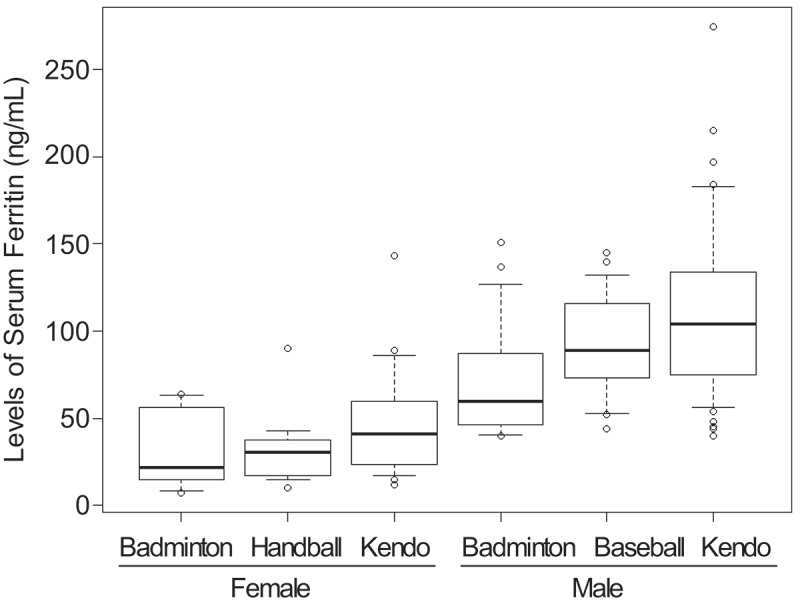
Serum ferritin levels classified by sex and affiliated club.

As an exploratory analysis, we investigated the distribution of serum ferritin levels classified by sex and sports clubs (Figure 1). The medians of serum ferritinemia in the badminton club were lower than the others in females and males; however, sex and sports clubs were not identified as a predictor of hypoferritinemia (*p* with two-way analysis of variance = 0.17 and 0.57, respectively).

Distribution of serum ferritin levels was shown after classifying by sex and the affiliated clubs. The line within box, box, whisker and dots indicate the median, interquartile range, 1.5 times the interquartile range and the outliers, respectively.

### 3.3. Characteristics associated with hypoferritinemia in female athletes

We then focused on female athletes since hypoferritinemia was observed in females only. The univariate analysis revealed the previous use of iron supplementation as a variable associated with hyperferritinemia (Table 3). Participant characteristics of age, experienced years of athletics, affiliated club, BMI, energy intake, and iron intake were further included in the multivariate logistic model as we removed a history of anemia and previous use of oral iron supplements due to potent confounders. The multivariate model (*Multivariate model 1*) identified shorter years of athletes (adjusted odds ratio [95% confidence interval]: 0.60 [0.42 − 0.87]), badminton club (358.4 [2.5 − 5.1 × 10^4^]), higher BMI (1.66 [1.01 − 2.71]), lower energy intake (0.99 [0.990 − 0.999]), and higher iron intake (4.90 [1.34 − 17.98]) were associated with hypoferritinemia. We then excluded the badminton club members from the logistic model due to the potential small-sample bias (“*Multivariate model 2*”). The shorter years of athletes (0.62 [0.43–0.90]), lower energy intake (0.994 [0.989 − 0.999]), and higher iron intake (4.40 [1.12 − 17.26]) kept the association with hypoferritinemia. The BMI, however, lost statistical significance in the revised regression model.

**Table 3.** Participant characteristics associated with hypoferritinemia in female athletes.

Variables	Hypo-ferritinemia (*n* = 22)	Normo-ferritinemia (*n* = 25)	Odds ratio of univariate analysis	*p* value	Multivariate Model 1		Multivariate Model 2	
					Adjusted odds ratio	*p* value	Adjusted odds ratio	*p* value
Age (years)	19 [19,20]	20 [19–21]	0.77 [0.44 − 1.35]	0.36	0.68 [0.28 − 1.63]	0.38	0.82 [0.34 − 2.01]	0.67
Years of sports/athletics	9 [7–13]	13 [11–15]	0.86 [0.74 − 1.00]	0.053	0.60 [0.42 − 0.87]	0.007	0.62 [0.43 − 0.90]	0.01
Affiliated club								
Kendo	8 (36)	12 (48)	1 (reference)		1 (reference)		1 (reference)	
Handball	10 (45)	10 (40)	1.50 [0.43 − 5.25]	0.44	0.52 [0.05 − 4.95]	0.57	0.77 [0.08 − 7.45]	0.77
Badminton	4 (18)	3 (12)	2.00 [0.35 − 11.44]	0.53	358.4 [2.5 − 5.1×10^4^]	0.02	−	−
Body mass index (kg/m^2^)	23.2 [21.9–24.8]	23.1 [21.7–24.1]	1.01 [0.79 − 1.28]	0.96	1.66 [1.01 − 2.71]	0.045	1.55 [0.94 − 2.56]	0.08
History of anemia*	4 (18)	1 (4)	0.31 [0.42 − 15.55]	0.31	−	−	−	−
Previous use of oral iron supplement*	13 (59)	7 (29)	3.71 [1.10 − 12.56]	0.03	−	−	−	−
FFQg survey^†^								
Dietary energy intake (kcal)	1,984 [1],479- [2],200]	1,835 [1],709- [2],376]	1.00 [0.99 − 1.00]	0.86	0.99 [0.99 − 0.999]	0.02	0.994 [0.989 − 0.999]	0.04
Dietary iron intake (mg)^‡^	6.6 [5.0–7.7]	5.9 [5.0–8.4]	1.04 [0.76 − 1.41]	0.82	4.90 [1.34 − 17.98]	0.02	4.40 [1.12 − 17.26]	0.03

Median [interquartile range] or number (percentage) are shown. *A female in the normo-ferritinemia group was missed. History of anemia and previous use of oral iron supplement were not included in the multivariate logistic model due to collinearity. ^†^Three and two females were missed in hypo- and normo-ferritinemia groups. The multivariate model 2 excluded the badminton club due to the potential small-sample bias.

Abbreviations: aOR; adjusted odds ratio, FFQg; food-frequency questionnaire based on food groups, OR; odds ratio. ^‡^Fe intake is defined as dietary intake.

### 3.4. Supplemental survey on the menstruation and usage of low-dose estrogen-progestin in female athletes

Thirty-four female athletes (72% of females) further responded to questionnaires on their menstruation and usage of low-dose estrogen-progestin (LEP) preparation (Supplemental Table 1). No females had a history of amenorrhea, while 16 (47%) had irregular menstruation. The fatigue and a decline in the subjective performance of the competition during menstruation were seen in 21 (62%) and 17 (50%) females, respectively, whereas only two (6%) and three (9%) females took LEP agents and had an experience of adjusting the menstrual period with LEP. There were no significant differences in the responses to the survey between female athletes with and without hypoferritinemia (Supplemental Table 2). We asked both male and female athletes about the knowledge of LEP for adjusting the menstrual cycle: 29 (85%) females knew it, although 34 out of 59 (58%) males did (*p* < 0.01).

## 4. Discussion

We demonstrated here that iron-deficient but non-anemic (IDNA) female athletes are prevalent as 47% (95% CI: 32–62) with serum ferritin levels <30 µg/L. Iron deficiency was commonly observed across sports clubs, including kendo, handball, and badminton. This result indicates that the evaluation of iron deficiency is important in the education of female athletes.

The final multivariate regression model exhibited significant associations between years as an athlete and energy and iron intakes with hypoferritinemia in females. The longer athletic career might prevent hypoferritinemia potentially due to educational interventions during their training program [32]. Dietary energy intake was inversely related to hypoferritinemia, while iron intake showed a positive correlation in this study. These observations diverge from prior research. Insufficient energy intake can contribute to low iron status in athletes [33,34]. A higher intake of dietary iron should be recommended to prevent anemia in athletes [32]. Unidentified confounding factors might address the contrary findings. One explanation could be the inhibition of dietary iron absorption in the gastrointestinal tract. Non-heme iron exhibits lower bioavailability compared to heme iron [35]. The lack of evaluation of dietary heme iron is a limitation of the present study. Medical histories of Helicobacter pylori infection and atrophic gastritis should also be determined in future studies on the ID status [36]. Another consideration is the inflammatory status related to their exercise, which can stimulate hepcidin production [37]. The elevated levels of serum hepcidin, in turn, can inhibit iron absorption. Energy deficiency and low carbohydrate diet may induce elevated levels of hepcidin [38]. Elucidating the relationship between the amount of exercise, serum hepcidin levels, and hypoferritinemia is crucial for understanding the biological and physiological background of ID status. Unfortunately, this study was limited by the absence of hepcidin measurements in our contracted laboratory. Of note, the higher BMI was associated with hypoferritinemia in the initial multivariate regression analysis but not in the revised model. This discrepancy suggests a confounding relationship between BMI and badminton club membership, which was removed due to the limited size of the cohort. Several studies reported a correlation between BMI, iron intake, and ferritin levels in athletes [35]. For instance, a multivariate analysis with 30 Canadian college female athletes revealed a positive correlation between BMI and ferritin levels [14]. Given these complexities, there is a clear need for more in-depth studies that assess detailed nutritional profiles, quantify exercise levels, and evaluate iron metabolism-related biomarkers, including hepcidin.

In our study, only two female athletes consistently used LEP, despite numerous female athletes reporting fatigue during menstruation and a perceived decrease in performance. Hormonal contraceptives have been widely used among young elite athletes: 57% of elite female athletes in Denmark [39], 32.7% of female football players in Australia [40], 63% of a variety of sports athletes in Nordic countries [31] reported usual use of hormonal contraceptives. While LEP or other hormonal contraceptives contribute to better performance by managing the menstrual cycle, there are known side effects such as mood changes, stomach pain, and migraines [39,41]. Some studies show LEP use during adolescence prevents bone mass acquisition [42]. It is necessary to be more educated about the effect of regular oral administration of LEP on performance and menstrual movement, including male athletes who will become leaders in the future.

There are several limitations in this study. First, there is a bias by the nature of a single-center observational study. The selection of sports clubs was not comprehensive. The absence of a control group of non-athlete university students prevented the identification of findings specific to athletes. Secondly, this study did not assess serum hepcidin levels, a known inhibitor of iron absorption due to exercise-induced inflammatory response. As a result, a comprehensive understanding of iron metabolism remains elusive. While exercise amount might be associated with hepcidin levels, we could not evaluate this potential correlation. Thirdly, the study did not distinguish between dietary heme and non-heme iron sources, which could potentially influence the outcomes of the multivariate regression models. Fourth, there was no data on the menstruation cycle at the time of blood testing. The menstruation cycle can influence both complete blood counts and ferritin levels. We surveyed the menstrual status and the awareness and use of LEP using a binary yes or “no” response format, which might develop biased answers. A scale-based response might capture precise insights. Similarly, the survey on iron supplements was the binary-response format, which limits in-depth analysis of the influence of the dosage and concomitant vitamin intake. Fifth, the small-sample bias should be treated in the large-cohort study in the future. The expansive 95% CI range emphasizes this need noted for the badminton club in the initial multivariate analysis (“*Multivariate model 1*”).

## 5. Conclusions

This study revealed that ID has been a common health issue in female university athletes, as 47% (95% CI: 32–62) of them showed hypoferritinemia across sports clubs in Japan. The findings call for enhanced management of iron status in young athletes. Limitations in this study include the nature of the single-site observational study and the absence of serum hepcidin measurement. These limitations restrict understanding of the biological changes in iron metabolism and its association with dietary and training profiles. More comprehensive studies are warranted to determine iron metabolism, dietary characteristics, and athletic performance, aiming to identify the optimal interventions to address iron deficiency in university athletes.
